# N-terminal-pro-brain natriuretic peptide is decreased in insulin dependent gestational diabetes mellitus: a prospective cohort trial

**DOI:** 10.1186/1475-2840-10-28

**Published:** 2011-04-13

**Authors:** Martin Andreas, Harald Zeisler, Ammon Handisurya, Maximilian B Franz, Michael Gottsauner-Wolf, Michael Wolzt, Alexandra Kautzky-Willer

**Affiliations:** 1 Department of Clinical Pharmacology, Medical University of Vienna, Austria; 2 Department of Obstetrics and Gynaecology, Medical University of Vienna, Austria; 3 Department of Internal Medicine III, Division of Endocrinology and Metabolism, Medical University of Vienna, Austria; 4 Department of Internal Medicine II, Division of Cardiology, Medical University of Vienna, Austria

**Keywords:** pregnancy, NT-proBNP, preeclampsia, GDM

## Abstract

**Background:**

N-terminal-pro-brain natriuretic peptide (NT-proBNP) is elevated in gestational hypertension and preeclampsia. This trial aimed to generate data for gestational diabetes mellitus patients, who are at risk to develop these complications.

**Methods:**

We have measured NT-proBNP in 223 otherwise healthy women between gestational week 24 and 32 referred to the outpatient diabetes unit in a cross-sectional study.

**Results:**

88 control subjects, 45 patients with indication for medical nutrition therapy (MNT) alone and 90 patients who required insulin therapy were included. Groups of women were comparable regarding gestational week. Body mass index before pregnancy and at blood draw was significantly higher in subjects with insulin dependent gestational diabetes mellitus compared to MNT controlled gestational diabetes mellitus. NT-proBNP was significantly lower in patients with insulin dependent gestational diabetes mellitus (35 ± 25 pg/ml) compared to controls (53 ± 43 pg/ml, p = 0.012).

**Conclusions:**

NT-proBNP is within the reference range of normal subjects in women with gestational diabetes mellitus. Differences in body mass index, changes in glomerular filtration rate and haemodynamics may explain lower NT-proBNP concentrations in insulin dependent gestational diabetes mellitus. A false negative interpretation needs to be considered in these women.

## Background

Women with gestational diabetes mellitus (GDM) are at risk to develop preeclampsia and other complications during pregnancy[1-3]. Previously, it has been reported that NT-proBNP is elevated in gestational hypertension and preeclampsia[4], but no data exist in GDM patients.

Amino-terminal pro B-type natriuretic peptide (NT-proBNP) is co-secreted with B-type natriuretic peptide (BNP) from the cardiac ventricle. It is increased in response to ventricular volume expansion and pressure overload[5,6]. The cardiovascular action of BNP includes vasodilation, diuresis, inhibition of renin and aldosterone production and reduction of cardiac and vascular growth[7,8]. Beside these direct pharmacological effects, BNP is also used as a biomarker in patients with heart failure to assess systolic ventricular dysfunction[9].

Although NT-proBNP reference values are well established in a healthy population, co-morbidities or altered physiological states may influence its plasma concentration. This is of particular interest in subjects with diabetes who are at increased cardiovascular risk. However, NT-proBNP reference ranges across patients with diabetes without acute complications are not appropriately studied.

In the present prospective cohort study, we have therefore measured circulating concentrations of NT-proBNP in women with different severity of GDM to evaluate if established reference ranges of this biomarker may also be used in this population.

## Methods

In a prospective cohort study, all pregnant women between gestational week 24 and 32 referred to the outpatient diabetes unit for the performance of an oral glucose tolerance test (oGTT) were included. The study protocol was approved by the Ethics Committee of the Medical University of Vienna. Informed consent was obtained and medical history taken. Inclusion criteria comprised age ≥18 years and singleton pregnancy. Exclusion criteria were presence of a clinically relevant disease other than GDM and intake of concomitant medication other than vitamin/iron supplementation.

All women with GDM received medical nutrition therapy (MNT). Insulin substitution was initiated independently from inclusion in the study according to current guidelines after one week of MNT[10]. MNT was sufficient in 45 women (MNT-GDM group) and additional insulin therapy was prescribed in 90 women (iGDM group) in order to maintain normoglycemia (fasting glucose < 95 and postprandial [1 h after meals] glucose < 130 mg/dl). No oral antihyperglycemic medication was used. A control group of 88 women with normal glucose tolerance was also included. Pregnancy outcome was followed up until eight weeks after childbirth, subjects with signs of any other pregnancy associated disease were excluded.

A blood draw was performed at 8:00 in the morning after an overnight fast of 12 hours. NT-proBNP was analysed using an immunoassay (ELECSYS^® ^proBNP, Roche Diagnostics GmbH, Mannheim, Germany) using an ELECSYS^® ^2010 instrument.

### Statistical analysis

Data sets were descriptively analysed and presented as means ± SD. For comparison of data, sets were tested for normal distribution. To compare outcome parameters between groups, an analysis of variance or the Kruskal-Wallis test were applied for parametric and non-parametric datasets, respectively. The Spearman correlation coefficient was calculated to analyse relationships. p-values lower than 0.05 were considered statistically significant. Statistical analysis was performed with the SPSS V14.0.1 (SPSS Inc., Chicago, Illinois, USA).

## Results

223 pregnant women were included in the study. Women with MNT-GDM or iGDM were comparable with controls regarding gestational week. MNT-GDM women were slightly younger than controls and iGDM patients. Body mass index (BMI) before pregnancy and at blood draw was significantly higher in subjects with iGDM (26.2 ± 5 and 29.9 ± 4.8 kg/m^2^) as compared to those with MNT-GDM (22.6 ± 3.9 and 26.2 ± 3.9 kg/m^2^; p < 0.005 and p < 0.001, respectively).

NT-proBNP was significantly lower in patients with iGDM as compared to controls (p = 0.012, Table 1). No differences were observed regarding haematocrit, total protein, albumin, creatinine, urinary glucose, urinary protein, C-reactive protein or thyroid-stimulating hormone between the groups (data not shown).

**Table 1:** Characteristics of pregnant women with MNT-GDM, iGDM, or normal glucose tolerance

	MNT-GDM	iGDM	normal
n	45	90	88
Age (years)	29 ± 5 P = 0.034*	32 ± 5 *p = 0.046*^*†*^	32 ± 6
Gestational week	28 ± 5	28 ± 5	27 ± 4
Fasting glucose (mg/dl)	84 ± 13	91 ± 14 p < 0.001* p = 0.012^*†*^	75 ± 10
oGTT 60 Min (mg/dl)	187 ± 28 p < 0.001*	188 ± 33 p < 0.001*	138 ± 33
oGTT 120 Min (mg/dl)	137 ± 25 p < 0.001*	143 ± 26 p < 0.001*	104 ± 22
HbA1c (%)	4.9 ± 0.3	5.1 ± 0.4 P = 0.02*	4.8 ± 0.5
HbA1c (mmol/mol)	30 ± 3	32 ± 4 P = 0.02*	29 ± 5
NT-proBNP (pg/ml)	46 ± 40	35 ± 25 P = 0.012*	53 ± 43

Data are means ± SD. GDM: gestational diabetes mellitus; MNT: medical nutrition therapy; iGDM: insulin dependent GDM; oGTT: oral glucose tolerance test; NT-proBNP: N-terminal pro brain natriuretic peptide; HbA1c: glycosylated hemoglobin; * p-value versus normal pregnancies; ^† ^p-value versus MNT-GDM

There was a negative correlation between NT-proBNP and HbA1c (r: -0.239; p: 0.004), total protein (r: -0.182; p: 0.025), haematocrit (r: -0.194; p: 0.014) and BMI before pregnancy (r: -0.363; p: < 0.001) and at blood draw (r: -0.262; p: < 0.001) in a pooled analysis of all subjects.

## Discussion

The principal finding of this study is that NT-proBNP is not elevated in women with GDM and that upper cut-off values may therefore also be applied to this group of patients. However, in the subgroup of women with iGDM, circulating NT-proBNP concentrations are lower than those seen in women with MNT-GDM or in healthy pregnancies. It is therefore possible that this biochemical marker of heart failure may not be sensitive enough to detect early signals of impaired cardiac function when similar reference ranges are applied. This risk of a false negative interpretation of laboratory results needs to be considered in these women at risk.

Our data are at variance with other studies reporting on higher NT-proBNP in subjects with type 2 diabetes mellitus[11-13]. Comparison of those datasets, however, is difficult because of the pathophysiology of long-standing diabetes as well as differences in age, renal function, gender and metabolic control. Interestingly, the influence of age also seems to be important in type 2 diabetic subjects, since lower NT-proBNP concentrations were observed in patients younger than 45 years when compared to controls[14]. This difference, however, did not persist in elderly subjects.

NT-proBNP was recently identified as a screening parameter for left ventricular diastolic dysfunction by Magnusson et al.[13]. About 50% of asymptomatic patients with type 2 diabetes mellitus showed left ventricular diastolic dysfunction. The onset of mild and severe left ventricular diastolic dysfunction correlated with increased NT-proBNP plasma levels. This effect was pronounced in female patients. Risk factors, underlying mechanisms and therapeutic options for left ventricular diastolic dysfunction are still not clear. Therefore, the occurrence of this pathology together with GDM should be addressed in further research.

As a limitation of this cross-sectional study, physiological cardiovascular changes that occur during pregnancy were not considered. It is known that peripheral vascular resistance is highest at the beginning of pregnancy and decreases after the 24^th ^week of gestation[15]. NT-proBNP is elevated in early pregnancy but returns to pre-pregnant levels prior to the 24^th ^week of gestation[16]. There is preliminary evidence from a small study in women with GDM that left ventricular mass may be augmented despite normal blood pressure[17]. This indicates that cardiovascular alterations exist in GDM which are not detected during routine screening.

GDM patients are at an increased risk of developing preeclampsia. NT-proBNP is know to be elevated in subjects with mild and severe preeclampsia, probably reflecting ventricular stress[18]. The prognostic value of increased NT-proBNP during pregnancy for preeclampsia has to be investigated in further projects. In addition, NT-proBNP is a valuable marker in patients with established heart disease prior to pregnancy[19]. The influence of GDM on NT-proBNP in these conditions has to be taken into account.

Serum creatinine was not different between study groups. However, glomerular filtration rate was not measured directly. Therefore, an influence of renal excretion on NT-proBNP variations in iGDM can not be excluded. Again, these relationships may vary during the course of pregnancies with or without GDM due to glomerular hyperfiltration and other haemodynamic changes.

The inverse relationship between NT-proBNP and BMI is in agreement with previous results from obese subjects[20] and suggests that differences in BMI between groups might have contributed partly to the reduction of NT-proBNP in iGDM. This study does also support an inverse relationship between NT-proBNP and haematocrit[21], but failed to detect an association with C-reactive protein (r:- 0.15, p = 0.07)[22].

## Conclusions

In conclusion, NT-proBNP reference ranges may be applied to women with GDM, but borderline values in women with insulin dependent GDM should be interpreted with caution and may be related to early haemodynamic changes.

## Competing interests

The authors declare that they have no competing interests.

## List of abbreviations

BMI: Body mass index; BNP: B-type natriuretic peptide; GDM: gestational diabetes mellitus; HbA1c: glycosylated haemoglobin molecule, haemoglobin A1C; iGDM: insulin dependent gestational diabetes mellitus; MNT: medical nutrition therapy; NT-proBNP: N-terminal-pro-brain natriuretic peptide, oGTT: oral glucose tolerance test; SD: standard deviation

## Authors' contributions

All authors fulfill the criteria for authorship. MA, MW and AK designed and drafted the protocol, HZ, MBF, MG and AH included subjects, performed clinical follow up and revised clinical data. MA, MW and AK wrote the final draft. All authors have read and approved submission of the final draft.

## References

[B1] Yasuhi I Hogan JW Canick J Sosa MB Carpenter MW Midpregnancy serum C-peptide concentration and subsequent pregnancy-induced hypertension Diabetes Care 2001 24 4 743 747 10.2337/diacare.24.4.743 11315841

[B2] Wolf M Sandler L Munoz K Hsu K Ecker JL Thadhani R First trimester insulin resistance and subsequent preeclampsia: a prospective study J Clin Endocrinol Metab 2002 87 4 1563 1568 10.1210/jc.87.4.1563 11932283

[B3] Sermer M Naylor CD Gare DJ Kenshole AB Ritchie JW Farine D Cohen HR McArthur K Holzapfel S Biringer A Impact of increasing carbohydrate intolerance on maternal-fetal outcomes in 3637 women without gestational diabetes. The Toronto Tri-Hospital Gestational Diabetes Project Am J Obstet Gynecol 1995 173 1 146 156 10.1016/0002-9378(95)90183-3 7631672

[B4] Tihtonen KM Koobi T Vuolteenaho O Huhtala HS Uotila JT Natriuretic peptides and hemodynamics in preeclampsia Am J Obstet Gynecol 2007 196 4 328 e321-327 10.1016/j.ajog.2006.11.033 17403408

[B5] Mukoyama M Nakao K Hosoda K Suga S Saito Y Ogawa Y Shirakami G Jougasaki M Obata K Yasue H Brain natriuretic peptide as a novel cardiac hormone in humans. Evidence for an exquisite dual natriuretic peptide system, atrial natriuretic peptide and brain natriuretic peptide J Clin Invest 1991 87 4 1402 1412 10.1172/JCI115146 1849149 PMC295184

[B6] Yasue H Yoshimura M Sumida H Kikuta K Kugiyama K Jougasaki M Ogawa H Okumura K Mukoyama M Nakao K Localization and mechanism of secretion of B-type natriuretic peptide in comparison with those of A-type natriuretic peptide in normal subjects and patients with heart failure Circulation 1994 90 1 195 203 8025996 10.1161/01.cir.90.1.195

[B7] Levin ER Gardner DG Samson WK Natriuretic peptides N Engl J Med 1998 339 5 321 328 10.1056/NEJM199807303390507 9682046

[B8] Hall C Essential biochemistry and physiology of (NT-pro)BNP Eur J Heart Fail 2004 6 3 257 260 10.1016/j.ejheart.2003.12.015 14987573

[B9] Mueller C Breidthardt T Laule-Kilian K Christ M Perruchoud AP The integration of BNP and NT-proBNP into clinical medicine Swiss Med Wkly 2007 137 1-2 4 12 17299662 10.4414/smw.2007.11614

[B10] Hadar E Hod M Establishing consensus criteria for the diagnosis of diabetes in pregnancy following the HAPO study Ann N Y Acad Sci 1205 88 93 10.1111/j.1749-6632.2010.05671.x 20840258

[B11] Magnusson M Melander O Israelsson B Grubb A Groop L Jovinge S Elevated plasma levels of Nt-proBNP in patients with type 2 diabetes without overt cardiovascular disease Diabetes Care 2004 27 8 1929 1935 10.2337/diacare.27.8.1929 15277419

[B12] Raymond I Groenning BA Hildebrandt PR Nilsson JC Baumann M Trawinski J Pedersen F The influence of age, sex and other variables on the plasma level of N-terminal pro brain natriuretic peptide in a large sample of the general population Heart 2003 89 7 745 751 10.1136/heart.89.7.745 12807847 PMC1767734

[B13] Magnusson M Jovinge S Shahgaldi K Israelsson B Groop L Melander O Brain natriuretic peptide is related to diastolic dysfunction whereas urinary albumin excretion rate is related to left ventricular mass in asymptomatic type 2 diabetes patients Cardiovasc Diabetol 9 2 10.1186/1475-2840-9-2 20078898 PMC2817679

[B14] Tildesley HD Aydin CM Ignaszewski A Strelzow JA Yu E Bondy G Sulfonylurea therapy is associated with increased NT-proBNP levels in the treatment of type 2 diabetes Int J Cardiol 2007 115 3 312 317 10.1016/j.ijcard.2006.03.014 16824633

[B15] Bosio PM McKenna PJ Conroy R O'Herlihy C Maternal central hemodynamics in hypertensive disorders of pregnancy Obstet Gynecol 1999 94 6 978 984 10.1016/S0029-7844(99)00430-5 10576186

[B16] Franz MB Andreas M Schiessl B Zeisler H Neubauer A Kastl SP Hess G Rhomberg F Zdunek D Maurer G NT-proBNP is increased in healthy pregnancies compared to non-pregnant controls Acta Obstet Gynecol Scand 2009 88 2 234 237 10.1080/00016340802596025 19096946

[B17] Oren S Golzman B Reitblatt T Turkot S Kogan J Segal S Gestational diabetes mellitus and hypertension in pregnancy: hemodynamics and diurnal arterial pressure profile J Hum Hypertens 1996 10 8 505 509 8895033

[B18] Seong WJ Kim SC Hong DG Koo TB Park IS Amino-Terminal Pro-Brain Natriuretic Peptide Levels in Hypertensive Disorders Complicating Pregnancy Hypertens Pregnancy 20701470 10.3109/10641950903115046

[B19] Tanous D Siu SC Mason J Greutmann M Wald RM Parker JD Sermer M Colman JM Silversides CK B-type natriuretic peptide in pregnant women with heart disease J Am Coll Cardiol 56 15 1247 1253 10.1016/j.jacc.2010.02.076 20883932

[B20] Bayes-Genis A DeFilippi C Januzzi JL Jr Understanding amino-terminal pro-B-type natriuretic peptide in obesity Am J Cardiol 2008 101 3A 89 94 10.1016/j.amjcard.2007.11.030 18243866

[B21] Muscari A Berzigotti A Bianchi G Giannoni C Ligabue A Magalotti D Sbano D Zacchini A Zoli M Non-cardiac determinants of NT-proBNP levels in the elderly: relevance of haematocrit and hepatic steatosis Eur J Heart Fail 2006 8 5 468 476 10.1016/j.ejheart.2005.10.018 16386463

[B22] Johnston N Jernberg T Lindahl B Lindback J Stridsberg M Larsson A Venge P Wallentin L Biochemical indicators of cardiac and renal function in a healthy elderly population Clin Biochem 2004 37 3 210 216 10.1016/j.clinbiochem.2003.11.002 14972643

